# The Effect of Word Frequency on Judgments of Learning: Contributions of Beliefs and Processing Fluency

**DOI:** 10.3389/fpsyg.2015.01995

**Published:** 2016-01-06

**Authors:** Xiaoyu Jia, Ping Li, Xinyu Li, Yuchi Zhang, Wei Cao, Liren Cao, Weijian Li

**Affiliations:** ^1^Department of Psychology and Behavioral Sciences, Zhejiang UniversityHangzhou, China; ^2^Department of Psychology, Zhejiang Normal UniversityJinhua, China; ^3^Institute of Developmental Psychology, Beijing Normal UniversityBeijing, China

**Keywords:** word frequency, judgments of learning, processing fluency, beliefs, cue-utilization framework

## Abstract

Previous research has shown that word frequency affects judgments of learning (JOLs). Specifically, people give higher JOLs for high-frequency (HF) words than for low-frequency (LF) words. However, the exact mechanism underlying this effect is largely unknown. The present study replicated and extended previous work by exploring the contributions of processing fluency and beliefs to the word frequency effect. In Experiment 1, participants studied HF and LF words and made immediate JOLs. The findings showed that participants gave higher JOLs for HF words than for LF ones, reflecting the word frequency effect. In Experiment 2a (measuring the encoding fluency by using self-paced study time) and Experiment 2b (disrupting perceptual fluency by presenting words in an easy or difficult font style), we evaluated the contribution of processing fluency. The findings of Experiment 2a revealed no significant difference in self-paced study time between HF and LF words. The findings of Experiment 2b showed that the size of word frequency effect did not decrease or disappear even when presenting words in a difficult font style. In Experiment 3a (a questionnaire-based study) and Experiment 3b (making pre-study JOLs), we evaluated the role of beliefs in this word frequency effect. The results of Experiment 3a showed that participants gave higher estimates for HF as compared to LF words. That is, they estimated that hypothetical participants would better remember the HF words. The results of Experiment 3b showed that participants gave higher pre-study JOLs for HF than for LF words. These results across experiments suggested that people’s beliefs, not processing fluency, contribute substantially to the word frequency effect on JOLs. However, considering the validation of the indexes reflecting the processing fluency in the current study, we cannot entirely rule out the possible contribution of processing fluency. The relative contribution of processing fluency and beliefs to word frequency effect and the theoretical implications were discussed.

## Introduction

Judgments of learning (JOLs), which refers to people’s prediction about the likelihood of remembering studied information, has been a core issue of investigation for researchers for over four decades (Koriat, 1997; Soderstrom and McCabe, 2011; Undorf and Erdfelder, 2011, 2015; Besken and Mulligan, 2013; Mueller et al., 2013, 2014). Given the significant impact of JOLs on regulating study time and appropriately allocating cognitive resources (for a review, see Dunlosky and Ariel, 2011), it is important to understand cues that influence JOLs and how people use them. Previous studies have confirmed that many cues and heuristics can influence JOLs (for a review, see Koriat, 2007), but the mechanisms underlying the effects of these cues are a core and current issue to researchers (Soderstrom and McCabe, 2011; Susser and Mulligan, 2014; Undorf and Erdfelder, 2015).

One intrinsic attribute of words that has had an effect on JOLs is word frequency (Begg et al., 1989; Benjamin, 2003). For instance, Begg et al. (1989, Experiment 1) examined whether people’s memory predictions for words with three levels of frequency (high, medium, and low) were different. Participants were asked to study these three kinds of words, rating the memorability and familiarity of them, respectively, on 7-point rating scales. The results revealed that participants judged high-frequency (HF) words as being more memorable and familiarity than low-frequency (LF) words, suggesting that word frequency affects JOLs. Benjamin (2003, Experiment 1) replicated the findings of Begg et al. (1989). Participants studied HF and LF words and made predictions of the likelihood of recognizing them later. The results showed that participants predicted higher rate of recognition for HF words than LF words, suggesting that the reliance on ease of fluency or familiarity made participants predict higher recognition performance for HF words during study. Susser and Mulligan (2014), however, failed to detect the word frequency effect on JOLs. In their experiment, they had participants write down HF and LF words with their dominant or non-dominant hand, and then make JOLs for each word. The results showed that word frequency did not affect JOLs, but the pattern was numerically in the expected direction. A possible reason is that hand-dominance manipulations make participants focus their attention on the subjective feeling of motoric fluency, which may interfere with the potential word frequency effect. Tullis and Benjamin (2012) proposed that word frequency effect on JOLs may be influenced by some uncontrolled factors. Hence, further research will be needed to provide more supportive evidence for this effect.

The underlying basis of the word frequency effect on JOL, however, is still not completely understood. According to the cue-utilization framework (Koriat, 1997), two hypotheses may account for the effect of word frequency on JOLs: the processing fluency hypothesis and the beliefs hypothesis. Processing fluency is the ease of processing items (Alter and Oppenheimer, 2009), while beliefs refer to any theories about how cues influence memory when making JOLs (Dunlosky et al., 2014; Mueller et al., 2015). These two hypotheses have been shown to contribute to the effects of many cues on JOLs (Dunlosky and Matvey, 2001; Koriat et al., 2004; Rhodes and Castel, 2008; Soderstrom and McCabe, 2011).

Koriat et al. (2004, p. 653) argued that “JOLs are based predominantly—perhaps exclusively—on the subjective experience associated with processing fluency.” With respect to the processing fluency hypothesis, people may make judgments by relying on online mnemonic cues, such as perceptual fluency (Rhodes and Castel, 2008, 2009; Besken and Mulligan, 2013), encoding fluency (Begg et al., 1989; Koriat and Ma’ayan, 2005), and retrieval fluency (Benjamin et al., 1998). The ease of processing leads to the subjective experience of familiarity and in turn unconsciously attributes to memorability (Dunlosky et al., 2014). Considering the word frequency effect on JOLs, Begg et al. (1989) explained that HF words were easier to process than LF words. This translated into an experience of fluency that leads to higher JOLs. However, no previous studies have examined the potential contribution of processing fluency to the word frequency effect via a direct manipulation.

An alternative hypothesis—the belief hypothesis—proposed that people may use their beliefs about memory to make JOLs (Koriat, 1997; Koriat et al., 2004; Mueller et al., 2014). These beliefs can exist prior to an experiment, be generated online to reduce uncertainty, or be developed across task experiences (Mueller et al., 2015). Dunlosky et al. (2014) converged the data about the relatedness, font size, and type format effects on JOLs, and found that these effects were mediated more by people’s beliefs. For example, Mueller et al. (2013, Experiment 1) had participants study related and unrelated pairs and make pre-study JOLs. The results showed that participants gave related pairs higher JOLs than unrelated pairs, suggesting the contribution of beliefs to the relatedness effect. Given that beliefs can be incorporated into JOLs (Koriat et al., 2004; Ariel et al., 2014), they can be used to explain the word frequency effect on JOLs. That is, people may have a belief that HF words are more memorable than LF words, and use it to guide their JOLs. Given that there has been little systematic study of the role of metacognitive beliefs on JOLs (Kornell and Bjork, 2009; Ariel et al., 2014), one goal of the present research is to help fill this significant gap in the literature.

Although both processing fluency and beliefs may account for the word frequency effect on JOLs, the degree to which they mediate the word frequency-JOLs relationship has not been directly investigated. Accordingly, we systematically investigated their relative contributions. In Experiment 1, we manipulated the cue of word frequency and attempt to replicate the word frequency effect on JOLs (Begg et al., 1989; Benjamin, 2003). Next, we directly evaluated the contribution of processing fluency to the word frequency effect. The amount of time (Koriat and Ma’ayan, 2005; Koriat, 2008; Undorf and Erdfelder, 2011, 2015; Mueller et al., 2014) participants spent in studying words (self-paced study time) was employed to reflect encoding fluency in Experiment 2a. A font manipulation of presenting words in a difficult font style (Song and Schwarz, 2008; Alter and Oppenheimer, 2009) was used to disrupt perceptual fluency in Experiment 2b. A questionnaire (Koriat et al., 2004; Mueller et al., 2014; Susser and Mulligan, 2014) on people’s prior beliefs about word frequency and memory was used in Experiment 3a and pre-study JOLs (Castel, 2008), which cannot be affected by processing fluency, were included in Experiment 3b. Both Experiments 3a and 3b were used to evaluate the contribution of beliefs to the word frequency effect on JOLs.

## Experiment 1

In Experiment 1, we manipulated word frequency (high versus low). Our prediction is that the word frequency has an impact on JOLs. Specifically, we predicted that participants would give higher JOLs for HF words than for LF words.

### Method

#### Participants

A total of 30 students (19 females) with an average age of 21.53 years (*SD* = 2.70), from Zhejiang Normal University took part in the study. They received partial credit for a course requirement or gifts, such as notebooks and pens.

The research procedure for Experiment 1 and subsequent experiments conform to the ethical standards of the 1964 Declaration of Helsinki. Zhejiang Normal University Review Board approved these research procedures and an informed consent form was signed by each participant prior to the experiment.

#### Design

The only within-participant variable was word frequency (high versus low).

#### Materials

A set of 158 Chinese words from the Modern Chinese Frequency Dictionary were chosen. We asked 31 independent raters to code the words in terms of their stroke number, the age of acquisition (7-point scale, 1 = 0–2 years; 7 = age 13 and over), familiarity (5-point scale, 1 = very strange; 5 = very familiar), and concreteness (5-point scale, 1 = very abstract; 5 = very specific). Based on the counterbalancing of these dimensions (*p*s > 0.05), we finally chose 16 HF words (from 185 to 603 occurrences per million) and 16 LF words (from 8 to 9 occurrences per million). Four additional words were presented as primacy and recency buffers at the beginning and end of the study list and excluded from all reported analyses.

#### Procedure

Each participant was tested individually on a computer. They were told to study 32 words for a later recall test. The experiment consisted of following phases:

Study and JOL phase: Participants studied each word for 2 s, with a 250-ms interval between each word. The order of words was randomly determined for each participant. However, two additional buffer words (one of each type) were always presented at the beginning and end of the list, respectively. Immediately after studying each word, participants made a self-paced JOL on it using six discrete response options, 0, 20, 40, 60, 80, 100 (0% = definitely did not remember the word; 100% = definitely remembered the word). These responses indicated the likelihood of recalling the word in a final recall test. The interval between the study and JOL of a word is minimal (Dunlosky and Nelson, 1997).

Test phase: After all words were studied and judged, participants completed a 3-min arithmetic task. Then, they took a self-paced recall test in which they wrote down as many studied words as they could remember in any order.

### Results and Discussion

**FIGURE 1:**
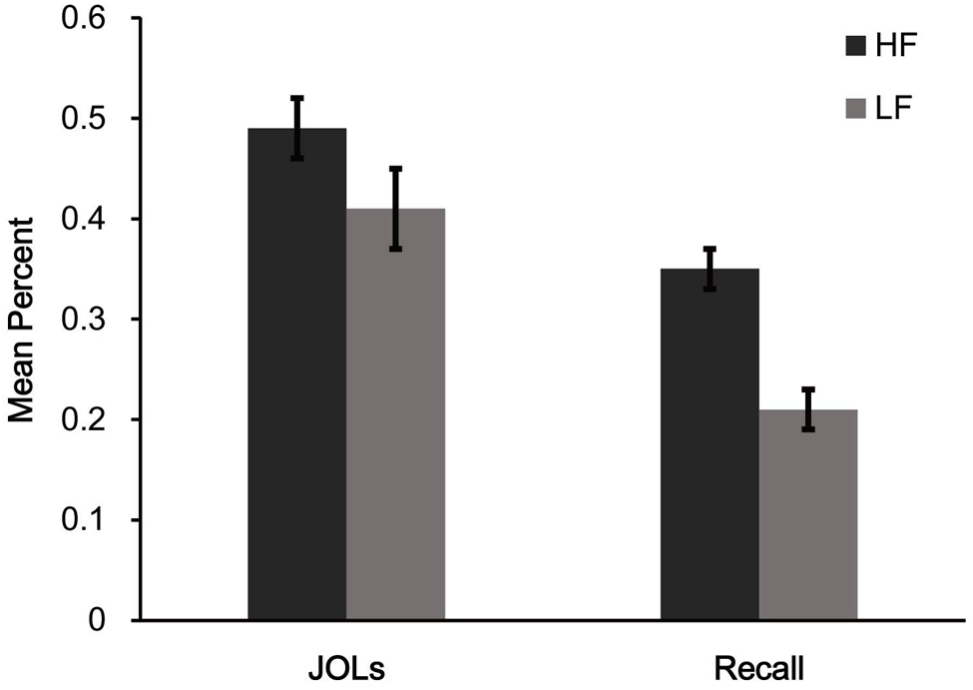
**Mean judgment of learning (JOL) and recall performance for different word frequency conditions in Experiment 1.** HF = high frequency words, LF = low frequency words. Error bars represent standard error of the mean.

Mean proportions of words correctly recalled (see **Figure 1**) were higher for HF words than for LF words. This was confirmed by a paired *t*-test between HF and LF words, *t*(29) = 4.47, *p* < 0.001, *d* = 1.11.

Mean JOLs (see **Figure 1**) showed that participants gave higher JOLs for HF words than for LF words, *t*(29) = 4.47, *p* < 0.001, *d* = 0.87. This result suggests that JOLs are affected by word frequency.

According to the cue-utilization framework (Koriat, 1997), JOLs can be based on the intrinsic cues because of their easily accessibility (Castel, 2008). That is, some inherent attributes of the studied materials could serve as an effective diagnostic of memorability and were used for people when making JOLs. For example, people used the degree of relatedness between pairs to make JOLs because it can disclose the ease or difficulty of learning or remembering those different pairs (Rabinowitz et al., 1982). Similarly, word frequency, one of the inherent attributes of the words, can be a cue for people to judge the difficulty of recalling different words, and thus affects JOLs.

## Experiment 2A

Encoding fluency refers to the ease with which items are committed to memory during study (Begg et al., 1989; Koriat and Ma’ayan, 2005). Previous studies used the amount of self-paced study time as an index of encoding fluency (Koriat and Ma’ayan, 2005; Koriat, 2008; Undorf and Erdfelder, 2011, 2015). The more study time an item requires, the less fluently it is encoded. The underlying assumption of it is that learners may use study time as a cue for JOLs, in line study time is inversely related to JOLs (Koriat et al., 2006). For example, Undorf and Erdfelder (2015) examined the relationship between self-paced study time and JOLs for related versus unrelated word pairs. Results revealed that, in accordance with the fact that JOLs were higher for related pairs than for unrelated pairs, mean self-paced study time were lower for related pairs than for unrelated pairs, suggesting that study time was used as a cue for JOLs by participants and processing fluency probably has mediated the relatedness effect on JOLs.

In Experiment 2a, we explored whether the encoding fluency, as measured by the amount of self-paced study time, contributes to the word frequency effect on JOLs. That is, the participants may use less time to study HF words than LF words and this study time difference may account for the word frequency effect on JOLs.

### Method

#### Participants

A total of 30 students (17 females) with an average age of 21.77 years (*SD* = 1.91) from Zhejiang Normal University participated in this study. In return, they received partial credit for a course requirement or gifts, such as notebooks and pens.

#### Design

The only within-participant variable was word frequency (high versus low).

#### Materials

The same materials were used as in Experiment 1.

#### Procedure

The procedure was the same as Experiment 1, with one exception. In this experiment, we used self-paced study time rather than a fixed-paced study time for each word. Participants were told that they could study each word as long as they needed and press the “space” key when they were through studying a word. They were also instructed that they should recall correctly as many words as they could remember in the final recall test.

### Results and Discussion

**FIGURE 2:**
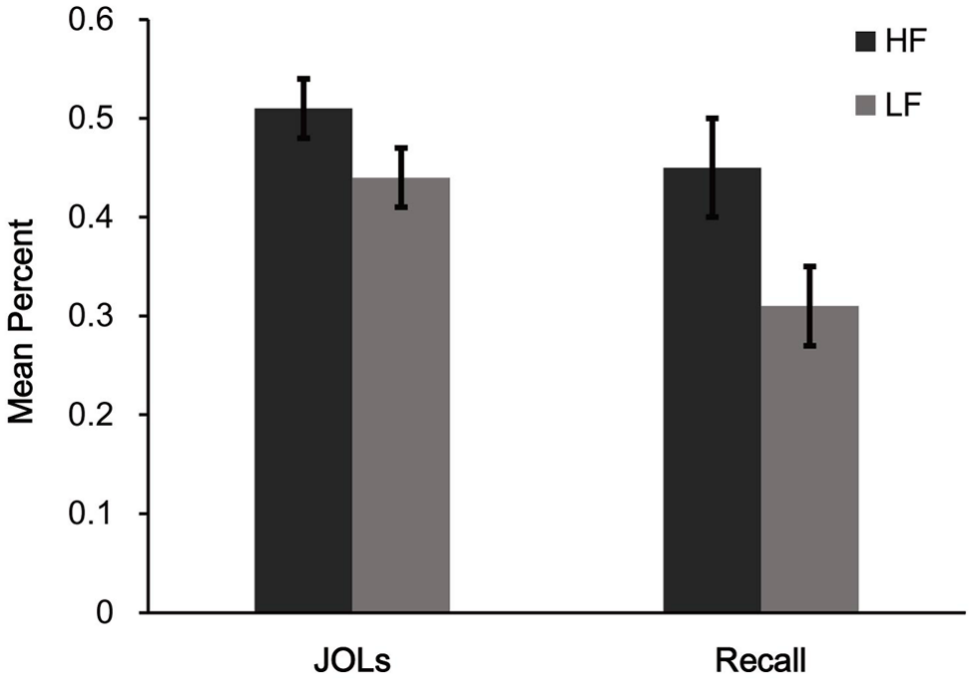
**Mean judgment of learning (JOL) and recall performance for different word frequency conditions in Experiment 2a.** HF = high frequency words, LF = low frequency words. Error bars represent standard error of the mean.

Mean proportions of words correctly recalled (see **Figure 2**) were higher for HF words than for LF words. This was confirmed by a paired *t*-test between HF and LF words, *t*(29) = 4.69, *p* < 0.001, *d* = 0.86.

Mean JOLs (see **Figure 2**) showed that participants gave higher JOLs for HF words than they did for LF words, *t*(29) = 5.29, *p* < 0.001, *d* = 1.04. Furthermore, a paired-samples *t*-test on self-spaced study time revealed no difference between HF words (*M* = 6.43 s, *SD* = 4.47) and LF words (*M* = 6.70 s, *SD* = 5.18), *t*(29) = –0.56, *p* = 0.58, *d* = 0.10.

To further assess whether encoding fluency mediates the relationship between word frequency and JOLs, a within-participant gamma correlation was computed for each participant, and then averaged across all participants (Koriat et al., 2006). The results showed that the correlation between word frequency and JOLs was.30, *t*(29) = 4.45, *p* < 0.001, *d* = 0.83, and that the correlation between word frequency and study time was –0.05 and non-significant, *t*(29) = –0.98, *p* = 0.34, *d* = 0.20. The correlation between JOLs and study time was –0.04 and also, non-significant, *t*(29) = –0.65, *p* = 0.52, *d* = 0.12. Most importantly, after controlling for study time, the correlation between word frequency and JOLs was 0.21, *t*(29) = 5.78, *p* < 0.001, *d* = 1.05, which did not differ from the zero-order correlation between word frequency and JOLs, *t*(29) = 0.33, *p* = 0.75, *d* = 0.17. Thus, study time did not mediate the relationship between word frequency and JOLs.

It was of surprise that Experiment 2a failed to reveal the significant negative correlation between self-paced study time and JOLs. Evidence from previous studies proposed that self-paced study time is the index of encoding fluency, which is determined by the word itself in a bottom-up fashion (Pelegrina et al., 2000; Koriat and Ma’ayan, 2005; Koriat et al., 2006). Thus, a negative correlation between self-paced study time and JOLs should be observed (Matvey et al., 2001; Koriat and Ma’ayan, 2005; Koriat et al., 2006; Undorf and Erdfelder, 2015). However, recent research suggests that the relationship between study time and JOLs could be influenced by other factors (Undorf and Erdfelder, 2011; Mueller et al., 2014), such as learning goals and item attributes. The researchers argued that when participants are explicitly instructed to make a JOL, they may adopt an analytic problem-solving mode (Analytic-Processing Theory, AP) to reduce uncertainty in their predictions (Mueller et al., 2015). That means the relationship between self-paced study time and JOLs may be goal-driven, not data-driven. For example, Mueller et al. (2014) found that the correlation between study time and JOLs was non-significant (*r* = 0.06; see also Mueller et al., 2015, *r* = –0.02 and non-significant). The possible reason was that the item attributes (i.e., font-size and identical pairs) had overshadowed the use of study time as a cue, and consequently influences JOLs. As for the results of Experiment 2a, the cuing effect of word frequency may overshadow the use of self-paced study time as a cue to influence JOLs. The non-significant relationship between self-paced study time and JOLs may be mediated by an analytic mode instead of a non-analytic mode of processing (Benjamin et al., 1998; Koriat et al., 2006; Koriat and Ackerman, 2010). If this was the case, the validity of self-paced study time as an index to measure processing fluency in the current study should be taken into consideration.

## Experiment 2B

Previous studies have demonstrated that perceptual fluency (i.e., the subjective experience of ease with which the stimulus is processed) substantially influence JOLs (Rhodes and Castel, 2008; Song and Schwarz, 2008; Alter and Oppenheimer, 2009; Forster et al., 2013). Reber et al. (2004) suggested that objective perceptual fluency manipulations, which vary the ease with which participants are able to perceive the stimuli (Alter and Oppenheimer, 2009), can feed into a subjective feeling of fluency and then influence JOLs. For example, Rhodes and Castel (2008) investigated whether items presented in standard or alternating formats would affect the font size effect on JOLs (i.e., participants gave higher JOLs for the larger size words, comparing with the smaller size ones). They found that disrupting perceptual fluency by presenting words in an alternating format diminished the font size effect, suggesting that perceptual fluency did play a role in the font size effect.

Similar to the type formats manipulation, font style manipulation, in which words are printed in either an easy font (e.g., Times New Roman) or a difficult font (e.g., a small gray, italicized font; Reber and Zupanek, 2002; Novemsky et al., 2007; West and Bruckmüller, 2013), has also been proved to be an effective way to disrupt subjective feeling of perceptual fluency by effects on experienced readability (Reber et al., 2004). The logic of the font style manipulation is that words presented in a difficult font style would disrupt participants’ perceptual fluency, which would in line compromise the influence of perceptual fluency on JOLs.

In Experiment 2b, we used a similar font style manipulation to alter the ease with which words could be read. If perceptual fluency played a role in the word frequency effect on JOLs, then this effect would decrease in magnitude or disappear when the words were presented in a difficult font style, comparing with an easy font style.

### Method

#### Participants

A total of 30 students (17 females) with an average age of 21.60 years (*SD* = 2.37) from Zhejiang Normal University participated in this study. For participation, they received gifts, such as notebooks and pens. None of them had previous taken part in a similar experiment.

#### Design

This experiment used a 2 (word frequency: high or low) × 2 (font style: easy or difficult) within-subjects design. For the easy font style, words were printed in Imitation Song, a bold font, such as eq001 (machine). In the difficult font style, the words were printed in Teng cheung, a similar bold, but also italicized font, such as **eq002** (girl). We evaluated the perceptual fluency of two kinds of font styles used in this experiment in a pretest. For each kind of font style, there was a word sample, which is different from the words presented in the subsequent experiment, presented to 21 independent raters, respectively. The raters were asked to rate the perceptual fluency of these two kinds of font styles on 5-point scale ranging from “very easy to read” to “very difficult to read,” which is similar to Novemsky et al. (2007). The result showed that words printed in the easy font style were much more easier to read (*M* = 1.05, *SD* = 0.22) than those printed in the difficult font style (*M* = 2.95, *SD* = 0.74), *t*(20) = –12.46, *p* < 0.001, *d* = 2.71.

#### Materials

The same materials were used in this experiment as in Experiment 1. Each set of 16 HF words and 16 LF words were randomly divided into two sub-sets of eight words, respectively. One sub-set was presented in the easy font and the other was presented in a different font. The four sub-sets were equated for stroke number, age of acquisition, familiarity, and concreteness (*p*s > 0.1). Two additional buffer words (one of each font styles) were always presented at the beginning and end of the list, respectively. They were excluded from all reported analyses.

#### Procedure

The procedure was the same as Experiment 1. However, half of the words were presented in an easy font and half were presented in a difficult font style.

### Results and Discussion

Mean proportions of words correctly recalled were computed. A 2 (word frequency: high or low) × 2 (font style: easy or difficult) ANOVA revealed a significant main effect for word frequency, *F*(1,29) = 35.03, *p* < 0.01, $\eta_{p}^{2}$ = 0.55. This indicated that participants’ recall performance was higher for HF words (*M* = 0.29, *SD* = 0.02) than for LF words (*M* = 0.15, *SD* = 0.02). There was no significant difference between recall performance of words presented in an easy font style (*M* = 0.20, *SD* = 0.03) and words presented in a difficult font style (*M* = 0.25, *SD* = 0.02), *F*(1,29) = 2.61, *p* = 0.12, $\eta_{p}^{2}$ = 0.08. The interaction between word frequency and font style was not significant, *F*(1,29) = 0.03, *p* = 0.88, $\eta_{p}^{2}$ = 0.001. In addition, the mean recall performance for HF words presented in an easy font style, HF words presented in a difficult font style, LF words presented in an easy font style, and LF words presented in a difficult font style were 0.26 (*SD* = 0.19), 0.32 (*SD* = 0.16), 0.13 (*SD* = 0.15) and.17 (*SD* = 0.15), respectively.

**FIGURE 3:**
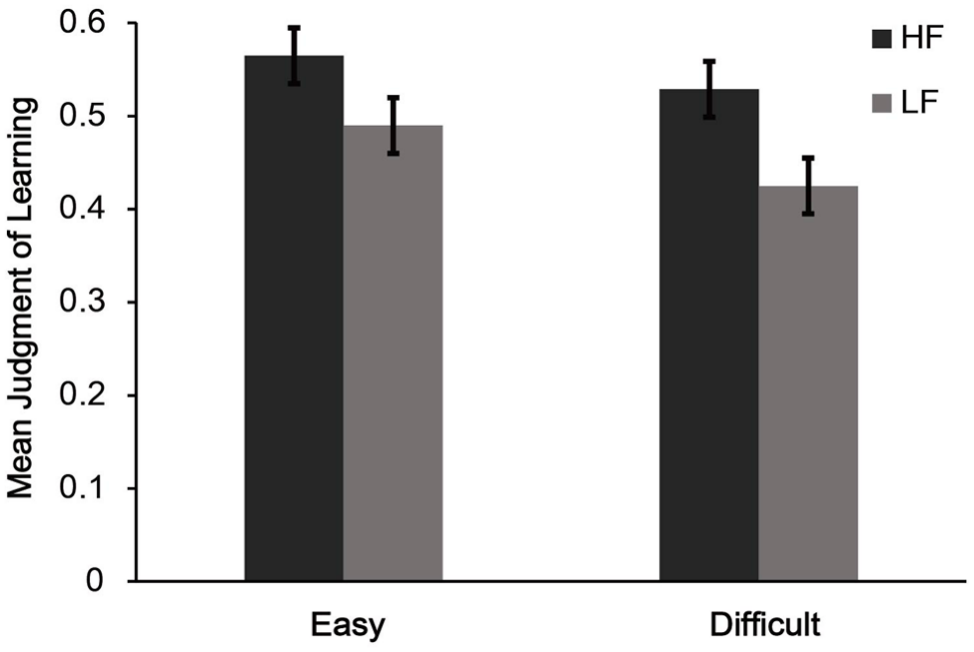
**Mean judgment of learning (JOL) for different word frequency and font styles conditions in Experiment 2b.** HF = high frequency words, LF = low frequency words; Easy = Easy font style, Difficult = Difficult font style. Error bars represent standard error of the mean.

A 2 (word frequency: high or low) × 2 (font style: easy or difficult) ANOVA on Mean JOLs (see **Figure 3**) revealed a significant main effect for word frequency, *F*(1,29) = 27.62, *p* < 0.01, $\eta_{p}^{2}$ = 0.49. This indicated that participants gave higher JOLs for HF words than for LF words. In addition, there was also a significant main effect of font style, *F*(1,29) = 4.55, *p* < 0.05, $\eta_{p}^{2}$ = 0.14, indicating that participants gave higher JOLs for words presented in an easy font than for words presented in a difficult font style. Most importantly, the interaction between word frequency and font style was not significant, *F*(1,29) = 2.60, *p* = 0.12, $\eta_{p}^{2}$ = 0.08, suggesting that differences in perceptual fluency have no contribution to the word frequency effect on JOLs.

However, we still should be cautious about this conclusion. The failure to report processing fluency contributing to word frequency effect may not be able to rule out the possibility of processing fluency mediating the word frequency effect on JOLs. Reber et al. (2004) suggested that font style manipulation could only disrupt the perceptual fluency of words, but not simultaneously disrupt their conceptual fluency, which means the ease of processing with words’ meaning and relatedness to other semantic knowledge structures. As the conceptual fluency difference between HF and LF words profoundly exists (Park et al., 2005), it may influence participants’ JOLs, even though the words were presented in a difficult font style. Namely, presenting HF and LF words in a difficult font might not completely eliminate the fluencies associated with processing LF and HF words and conceptual fluency may still in part mediate the relationship between word frequency and JOLs. Therefore, in the present study, it is still possible that processing fluency did contribute to word frequency effect notwithstanding that the difference in perceptual fluency did not appear to influence the word frequency effect on JOLs.

## Experiment 3A

In Experiment 3a, we adopted a standard questionnaire based method to examine the beliefs hypothesis (e.g., Koriat et al., 2004; Mueller et al., 2014, 2015). The questionnaire provides a scenario about an experiment, in which hypothesized students will study HF and LF words and later recall them. We asked the participants to evaluate the hypothesized students’ recall performance of different kinds of words in the described experiment and give the corresponding reasons. If the participants report that the hypothesized students would recall more HF words than for HF words, the beliefs hypothesis could be confirmed.

### Method

#### Participants

A total of 40 students (24 females) with an average age of 21.60 years (*SD* = 2.37) were recruited from Zhejiang Normal University to participate in this study. In return, they were given gifts, such as notebooks and pens.

#### Materials, Design, and Procedure

Participants read the following description of the experiment:

In a previous experiment, we asked some students to study 32 Chinese words. The frequency level of these 32 words was different. Half of the words were high frequency, which means that they appear often in our spoken and written language. In addition, half of the words were low frequency, which means that these words appear rarely in our spoken and written language. Each of the words was presented for 2 s on the screen and students were required to study them for an upcoming recall test. After studying all of the words, students completed a 3-min arithmetic task. Then, they were asked to write down as many of the words as they could remember in any order.

After reading the above description, participants estimated the number of each type of words that students would recall and wrote down the possible reasons. The order of the estimates for HF and LF words were counterbalanced across participants.

### Results and Discussion

A paired-sample *t*-test on estimation revealed a significant difference between HF words (*M* = 0.60, *SD* = 0.12) and LF words (*M* = 0.39, *SD* = 0.12), *t*(39) = 8.76, *p* < 0.001, *d* = 1.22, suggesting that people have an *a priori* belief about the effect of word frequency on memory.

As for the reasons that participants gave, we found that 87.5% participants (*n* = 35) thought that students would remember more HF words than LF words. Their reasons were as follows: (a) HF words are easier to remember (28.6% participants); (b) we communicate with and use more HF words in our life (51.4% participants); (c) HF words are easier to be activated and drawn from our memory (25.7% participants); (d) HF words are common (54.3% participants); (e) HF words are more associative and we remember them deeply (28.6% participants). Ten percent of participants (*n* = 4) indicated that LF words would be remembered more than HF words. They thought that students would pay more attention when studying LF words because of their novelty. Only one participant answered that students would remember the same amount of HF and LF words, since people have the same level of ability to remember both types of words. Overall, most people believed that HF words are more memorable than LF words and always drew on various reasons to support their ideas.

As suggested by Koriat et al. (2004) and Mueller et al. (2015), the method of questionnaire isolate theory-based influences on metamemory, because participants did not need to have the experience that students in the actual experiment had, so we can conclude that it is belief other than the subjective feeling of processing fluency that contributes to the word frequency effect on JOLs.

## Experiment 3B

In Experiment 3b, we used another method—pre-study JOLs to further explore the role of beliefs in word frequency effect on JOLs. Specifically, prior to studying each word, participants were prompted with the type of the word that would be presented next for study and were asked to make judgments for it. For example, Mueller et al. (2014, Experiment 4) asked participants to study large and small words and make pre-study JOLs for them. The results showed that participants gave large words higher JOLs than small words, suggesting the contribution of beliefs to the font size effect.

### Method

#### Participants

A total of 34 students (23 females) with an average age of 21.65 years (*SD* = 2.07) from Zhejiang Normal University participated in return for gifts, such as notebooks and pens.

#### Design

The only within-participant variable was word frequency (high versus. low).

#### Materials

The same materials were used in this experiment as the materials used in Experiment 1.

#### Procedure

The procedure for this experiment was the same as the procedure used in Experiment 1, except that we used pre-study JOLs instead of immediate JOLs. Before studying each word, participants were asked to make self-paced pre-study JOLs with the prompt: “You are about to study a HF/LF word (presented randomly). Please rate how likely you are to remember it.”

### Results and Discussion

**FIGURE 4:**
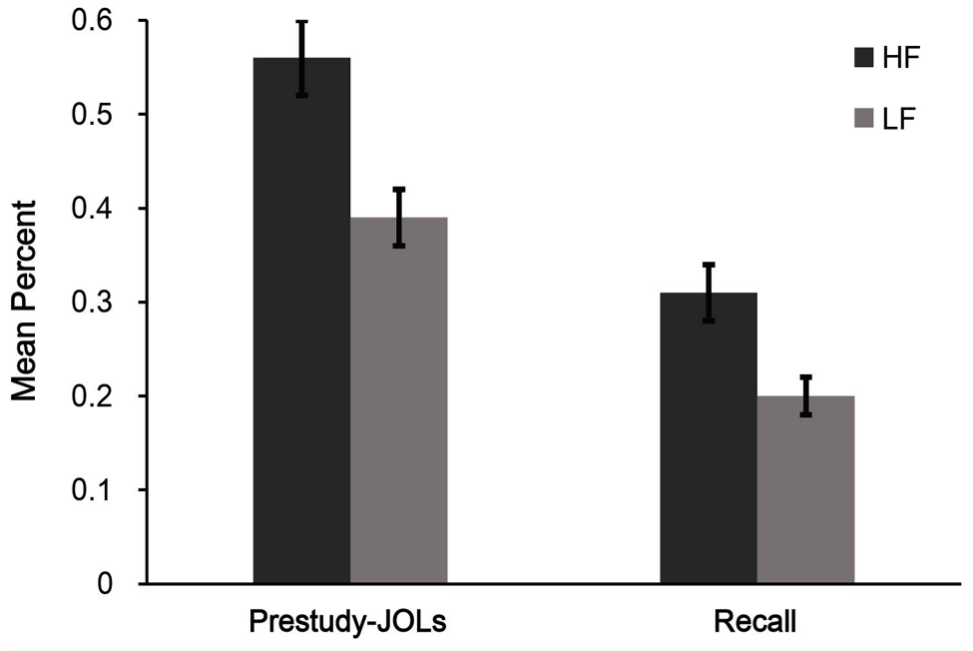
**Mean pre-study judgment of learning (pre-study JOL) and recall performance for different word frequency conditions in Experiment 3b.** HF = high frequency words, LF = low frequency words. Error bars represent standard error of the mean.

Mean proportions of words correctly recalled (see **Figure 4**) showed that participants gave higher for HF words than for LF words, *t*(33) = 5.05, *p* < 0.001, *d* = 0.93.

Mean pre-study JOLs (see **Figure 4**) showed that participants gave higher pre-study JOLs for HF words than for LF words, *t*(33) = 6.58, *p* < 0.001, *d* = 1.19. This confirmed the beliefs hypothesis that people draw on their beliefs about the memorability of HF words when making JOLs.

In addition, we examined the word frequency effect on pre-study JOLs via metamemory serial position analysis. Serial position analysis was constructed by computing the mean pre-study JOLs of four consecutive serial positions (i.e., position 1 = words 1–4, position 2 = items 5–8, etc.) as a function of word frequency. This analysis can enable us to test whether the word frequency effect on pre-study JOLs resulted from experience during the task or from applying beliefs (Castel, 2008; Tauber and Dunlosky, 2012; Mueller et al., 2014). If the task experience is responsible for the word frequency effect on JOLs, then the size of this effect may be initially small and increase across serial positions. The possibility is that HF words were more fluently processed than LF words, then experiencing this differential processing fluency across serial positions would increase the size of word frequency effect. However, If participants used beliefs to make JOLs, then the size of this effect may be parallel across serial positions. We divided 32 words into eight serial position bins (e.g., serial positions 1–4, 5–8, etc.) as a function of word frequency. A 2 (word frequency) × 8 (serial position bin) ANOVA revealed a main effect for word frequency, *F*(1,29) = 30.77, *p* < 0.001, $\eta_{p}^{2}$ = 0.52, indicating that participants made higher pre-study JOLs for HF words than for LF words. The main effect of serial position bin was significant, *F*(7,203) = 2.78, *p* < 0.01, $\eta_{p}^{2}$ = 0.09, with pre-study JOLs decreasing across serial position bins. The interaction between word frequency and serial position was not significant, *F*(7,203) = 1.40, *p* = 0.25, $\eta_{p}^{2}$ = 0.05. These results revealed that the word frequency effect on pre-study JOLs was partly resulted from participants’ relying on their beliefs about cues of word frequency.

Overall, both the results of pre-study JOLs and serial position analysis suggested that people’s beliefs contributed to the word frequency effect on JOLs. According to Jacoby and Kelley (1987), memory sometimes could be used as an object. When people were asking to make prediction for memory, they tried to search for the cues which can influence memory to guide their JOLs, in order to reduce their uncertainty. Similarly, when making pre-study JOLs prior to studying the upcoming word in current experiment, participants must use extrinsic information, such as the explicit instruction about the type of words, to guider their JOLs because of the absence of experiencing subjective feeling of processing fluency (Castel, 2008). In this case, the type of words can be thought to influence memory and participants used it to develop beliefs and in turn to guide JOLs (Dunlosky et al., 2014; Mueller et al., 2015).

## General Discussion

The goal of the current experiments was to explore the role of processing fluency and beliefs in mediating the relationship between word frequency and JOLs. In Experiment 1, we confirmed the existence of the word frequency effect on JOLs, where people gave higher JOLs for HF words than for LF words (Begg et al., 1989; Benjamin, 2003). In Experiment 2a, we found that there was no significant difference in self-paced study time between HF and LF words. We also found that self-paced study time did not mediate the relationship between word frequency and JOLs. In Experiment 2b, disrupting processing fluency by presenting words in a difficult font style did not decrease or eliminate the word frequency effect on JOLs. The results from both Experiments 2a and 2b do not support the processing fluency hypothesis. In Experiment 3, we found evidence in support of the beliefs hypothesis, both when using a questionnaire-based method (Experiment 3a) and when using a pre-study JOL method (Experiment 3b). These findings demonstrate that people use beliefs about the relation between word frequency and memory to make predictions.

According to dual-process models of JOLs (Koriat, 1997; Koriat et al., 2004), both beliefs and processing fluency can contribute to JOLs. For example, the relatedness effect on JOLs has been demonstrated to be mediated by both beliefs (Mueller et al., 2013, Experiment 1) and processing fluency (Undorf and Erdfelder, 2015, Experiments 1 and 2). As for the word frequency effect on JOLs, we found that beliefs played an important role in it. That is, people rely on the deliberate use of specific beliefs about the memorability of HF words to make predictions. The information we collected from our questionnaire reveals that HF words are perceived to be more common and relatively easier to remember, activate, and draw from memory. These beliefs may result from everyday experiences where HF words are more common and easier to remember, thereby influencing JOLs. This possible explanation is supported by Koriat et al. (2004, p. 644), who have stated that “people make use of their *a priori* theories about memory in making JOLs.” Nevertheless, participants might not have prior beliefs and these beliefs about word frequency could be developed on-line. Drawing on the analytic processing theory (Dunlosky et al., 2014; Mueller et al., 2015), when people make judgments about future memory, an analytic problem-solving mode could be triggered in which people tried to search for the cues which related to memory to reduce their uncertainty. Once the cues are thought to affect memory, they may be used to develop beliefs and participants apply the beliefs to improve the accuracy of JOLs (Dunlosky et al., 2014). For example, Mueller et al. (2015) manipulated the degree of semantic relatedness between the pairs (identical, related, and unrelated) to explore the influence of pairs relatedness to JOLs. The result showed that participants made higher JOLs for identical pairs than for related pairs. They further examined that this identical effect was mediated by beliefs, that is, participants thought that identical pairs were easier to remember and recall in the later test, and they developed the beliefs about how pairs relatedness affect memory when making JOLs. As for the current experiment, people presumably are looking for cues that indicate a word will be memorable at a later time. In this case, the explicit instruction about the type of words (HF or LF words) may make people think that different types of words affect memory. They used it as an available cue to develop beliefs and then applied the beliefs about how word frequency affects memory to make JOLs. Given that beliefs about how word frequency affects JOLs can develop in many ways, an important question for future research to investigate is whether these beliefs are produced before or during an experiment.

Although the current results provided little direct support for the processing fluency hypothesis, we still cannot rule out the possibility that processing fluency does mediate the word frequency effect. One explanation is that there might be other aspects of processing fluency which were not grasped by the current fluency measures could make a contribution, such as conceptual fluency (Alter and Oppenheimer, 2009; Mueller et al., 2014) and retrieval fluency (Benjamin et al., 1998; Koriat and Ma’ayan, 2005). For example, font style manipulation in Experiment 2b might have not disrupted the conceptual fluency of words and accordingly conceptual fluency still in part mediated the word frequency effect. Another explanation is that processing fluency may influence the word frequency effect indirectly through beliefs, in addition to the potentially direct influence of conceptual fluency. As suggested by Shah and Oppenheimer (2007), processing fluency can affect judgment in two ways. One is the direct way, in which the ease of processing itself acts as a piece of information and can be applied to a judgment. The other is the indirect way, in which the ease of processing might determine which cues are used to confront a task and these cues in turn affect judgment. For example, Serra and Dunlosky (2005) suggested that the negative relationship between retrieval latency and JOLs can be explained either by the non-conscious and direct influence of subjective experience of fluent retrieval, or by the indirect influence of beliefs about the retrieval latency affects recall. According to the AP theory (Dunlosky et al., 2014; Mueller et al., 2015), participants seek available cues which related to memory to reduce uncertainty about the future performance. In Experiment 2b, when words were presented from easy to difficult font, people may consciously monitor this variation and hold different expectations for the fluency of processing HF and LF words, as suggested by the discrepancy-attribution hypothesis (Whittlesea, 2002). In this case, the differential relative fluency between HF and LF words, which means the different level of discrepancy between expectations and on-going fluency of processing across HF and LF words (Jacoby and Dallas, 1981; Whittlesea and Leboe, 2003), could lead to different JOLs (Dunlosky et al., 2014). Namely, the differential relative processing fluency across words may lead people to consider that word frequency can affect memory. They then used the cue of word frequency to develop beliefs and applied the beliefs about how word frequency affects memory to make JOLs. If this is the case, the way processing fluency contribute to word frequency effect may be no longer unconsciously via a subjective feeling (Rhodes and Castel, 2008; Undorf and Erdfelder, 2011), but consciously through beliefs about how available cues affect memory (Dunlosky et al., 2014; Mueller et al., 2015). Future research should evaluate this possible joint contribution of beliefs and processing fluency empirically.

Several limitations of the present research should be noted. Firstly, the validity of self-paced study time as an index to measure processing fluency needs more supportive evidence. Considering that self-paced study time can be affected by a variety of factors (Mueller et al., 2015) and the degree of influence by these factors may decrease or overshadow the likelihood of mediation size of encoding fluency on word frequency effect, future research is needed to evaluate the validity of self-paced study time as an index of encoding fluency. Secondly, the contribution of processing fluency to the word frequency effect was probably underestimated. In Experiment 3b, before participants made a pre-study JOL for each word, the frequency level of word was presented. Thus, information on whether the upcoming word was HF or LF was available, but not with immediate JOLs. This may have led to a particularly pronounced influence of beliefs on pre-study JOLs. Thirdly, considering that the degree of processing fluency contributes to many cues effect on JOLs can be influenced by the systematic differences between different measures (Undorf and Erdfelder, 2015), future research is essential to use other established measures, which can real reflect processing fluency, to further reveal the role of processing fluency in the word frequency effect on JOLs, such as the number of trials needed for correct recall (Koriat, 2008; Undorf and Erdfelder, 2015) and the response time in a lexical decision task (Mueller et al., 2014).

## Conflict of Interest Statement

The authors declare that the research was conducted in the absence of any commercial or financial relationships that could be construed as a potential conflict of interest.
